# Supplementary material for: Diagnostic Performance of Biomarkers for Bladder Cancer Detection Suitable for Community and Primary Care Settings: A Systematic Review and Meta-Analysis
Source: Cancers (Basel). 2023 Jan 24;15(3):709. doi: 10.3390/cancers15030709 (PMC9913596; doi:10.3390/cancers15030709)

Supplementary Materials

Figure S1. PRISMA-DTA Checklist

PRISMA–DTA Checklist

Section/topic	#	PRISMA-DTA Checklist Item	Reported on page #
**TITLE / ABSTRACT**			
Title	1	Identify the report as a systematic review (+/- meta-analysis) of diagnostic test accuracy (DTA) studies.	
Abstract	2	Abstract: See PRISMA-DTA for abstracts.	
**INTRODUCTION**			
Rationale	3	Describe the rationale for the review in the context of what is already known.	
Clinical role of index test	D1	State the scientific and clinical background, including the intended use and clinical role of the index test, and if applicable, the rationale for minimally acceptable test accuracy (or minimum difference in accuracy for comparative design).	
Objectives	4	Provide an explicit statement of question(s) being addressed in terms of participants, index test(s), and target condition(s).	
**METHODS**			
Protocol and registration	5	Indicate if a review protocol exists, if and where it can be accessed (e.g., Web address), and, if available, provide registration information including registration number.	
Eligibility criteria	6	Specify study characteristics (participants, setting, index test(s), reference standard(s), target condition(s), and study design) and report characteristics (e.g., years considered, language, publication status) used as criteria for eligibility, giving rationale.	
Information sources	7	Describe all information sources (e.g., databases with dates of coverage, contact with study authors to identify additional studies) in the search and date last searched.	
Search	8	Present full search strategies for all electronic databases and other sources searched, including any limits used, such that they could be repeated.	
Study selection	9	State the process for selecting studies (i.e., screening, eligibility, included in systematic review, and, if applicable, included in the meta-analysis).	
Data collection process	10	Describe method of data extraction from reports (e.g., piloted forms, independently, in duplicate) and any processes for obtaining and confirming data from investigators.	
Definitions for data extraction	11	Provide definitions used in data extraction and classifications of target condition(s), index test(s), reference standard(s) and other characteristics (e.g. study design, clinical setting).	
Risk of bias and applicability	12	Describe methods used for assessing risk of bias in individual studies and concerns regarding the applicability to the review question.	
Diagnostic accuracy measures	13	State the principal diagnostic accuracy measure(s) reported (e.g. sensitivity, specificity) and state the unit of assessment (e.g. per-patient, per-lesion).	
Synthesis of results	14	Describe methods of handling data, combining results of studies and describing variability between studies. This could include, but is not limited to: a) handling of multiple definitions of target condition. b) handling of multiple thresholds of test positivity, c) handling multiple index test readers, d) handling of indeterminate test results, e) grouping and comparing tests, f) handling of different reference standards	

Section/topic	#	PRISMA-DTA Checklist Item	Reported on page #
Meta-analysis	D2	Report the statistical methods used for meta-analyses, if performed.	
Additional analyses	16	Describe methods of additional analyses (e.g., sensitivity or subgroup analyses, meta-regression), if done, indicating which were pre-specified.	
**RESULTS**			
Study selection	17	Provide numbers of studies screened, assessed for eligibility, included in the review (and included in meta-analysis, if applicable) with reasons for exclusions at each stage, ideally with a flow diagram.	
Study characteristics	18	For each included study provide citations and present key characteristics including: a) participant characteristics (presentation, prior testing), b) clinical setting, c) study design, d) target condition definition, e) index test, f) reference standard, g) sample size, h) funding sources	
Risk of bias and applicability	19	Present evaluation of risk of bias and concerns regarding applicability for each study.	
Results of individual studies	20	For each analysis in each study (e.g. unique combination of index test, reference standard, and positivity threshold) report 2x2 data (TP, FP, FN, TN) with estimates of diagnostic accuracy and confidence intervals, ideally with a forest or receiver operator characteristic (ROC) plot.	
Synthesis of results	21	Describe test accuracy, including variability; if meta-analysis was done, include results and confidence intervals.	
Additional analysis	23	Give results of additional analyses, if done (e.g., sensitivity or subgroup analyses, meta-regression; analysis of index test: failure rates, proportion of inconclusive results, adverse events).	
**DISCUSSION**			
Summary of evidence	24	Summarize the main findings including the strength of evidence.	
Limitations	25	Discuss limitations from included studies (e.g. risk of bias and concerns regarding applicability) and from the review process (e.g. incomplete retrieval of identified research).	
Conclusions	26	Provide a general interpretation of the results in the context of other evidence. Discuss implications for future research and clinical practice (e.g. the intended use and clinical role of the index test).	
**FUNDING**			
Funding	27	For the systematic review, describe the sources of funding and other support and the role of the funders.	

Adapted From: McInnes MDF, Moher D, Thombs BD, McGrath TA, Bossuyt PM, The PRISMA-DTA Group (2018). Preferred Reporting Items for a Systematic Review and Meta-analysis of Diagnostic Test Accuracy Studies: The PRISMA-DTA Statement. JAMA. 2018 Jan 23;319(4):388-396. doi: 10.1001/jama.2017.19163.

For more information, visit: www.prisma-statement.org.

Table S1. PICOS Framework - Inclusion & Exclusion Criteria

PICOS	Included	Excluded
Population	*Patients*:<br>• Adults >18yrs (individuals aged < 18 included only if outliers in large samples)<br>• Presenting with clinical signs and symptoms suggestive of bladder cancer (undergoing evaluation) (recurrence cases included only if outliers in large samples)<br>• N ≥ 50	*Patients*:<br>• Already diagnosed with bladder cancer<br>• Being monitored for recurrence<br>• With prior history of urinary cancers<br>• Paediatric populations<br>• N < 50
Intervention	Biomarker(s):<br>• Derived from human blood (serum, plasma), urine, faecal, salivary or breath samples.<br>• Tested individually or in panels/combinations<br>• In conjunction with urine cytology<br>• Validated in a population beyond the discovery/development phases	*Biomarker(s)*:<br>• No biomarkers - only urine Cytology investigated<br>• Validated in a population at discovery/development phases
Comparator(s)	*Non-cancer patients (controls):*<br>• Healthy individuals<br>• Symptomatic individuals<br>• Population with relevant non-malignant or pre-malignant conditions<br>• N ≥ 50	*Non-cancer patients (controls):*<br>• No non-cancer/ control group<br>• N < 50
Context	*Setting:*<br>• Any health care setting (primary, secondary, or tertiary)<br>• Any country	*Setting:*<br>• No restrictions
Outcome(s)	*Diagnostic performance/accuracy of biomarkers:*<br>• Reporting on at least one measure of diagnostic performance including sensitivity (SN), specificity (SP), positive predictive value (PPV), negative predictive value (NPV), and area under the curve (AUC)<br>*Suitability for Primary Care:*<br>• Reporting on effects on patients and clinicians	*Diagnostic performance/accuracy of biomarkers:*<br>• Reporting no measure of diagnostic performance

	including acceptability, feasibility, benefits, and harms if available<br>• Reporting on effects on diagnostic triage, the incorporation of tests(s) into diagnostic strategies and health economic data if available	
Study type	*Published Output:*<br>• Papers of any design in any language reporting original empirical findings published in peer-reviewed journals from 2000 onwards	*Published Output:*<br>• Book chapters, letters, comments, editorials, reviews<br>• Conference abstracts and proceedings<br>• Papers only reporting on animal or in-vitro models<br>• Before 2000

Figure S2. CanTest Framework

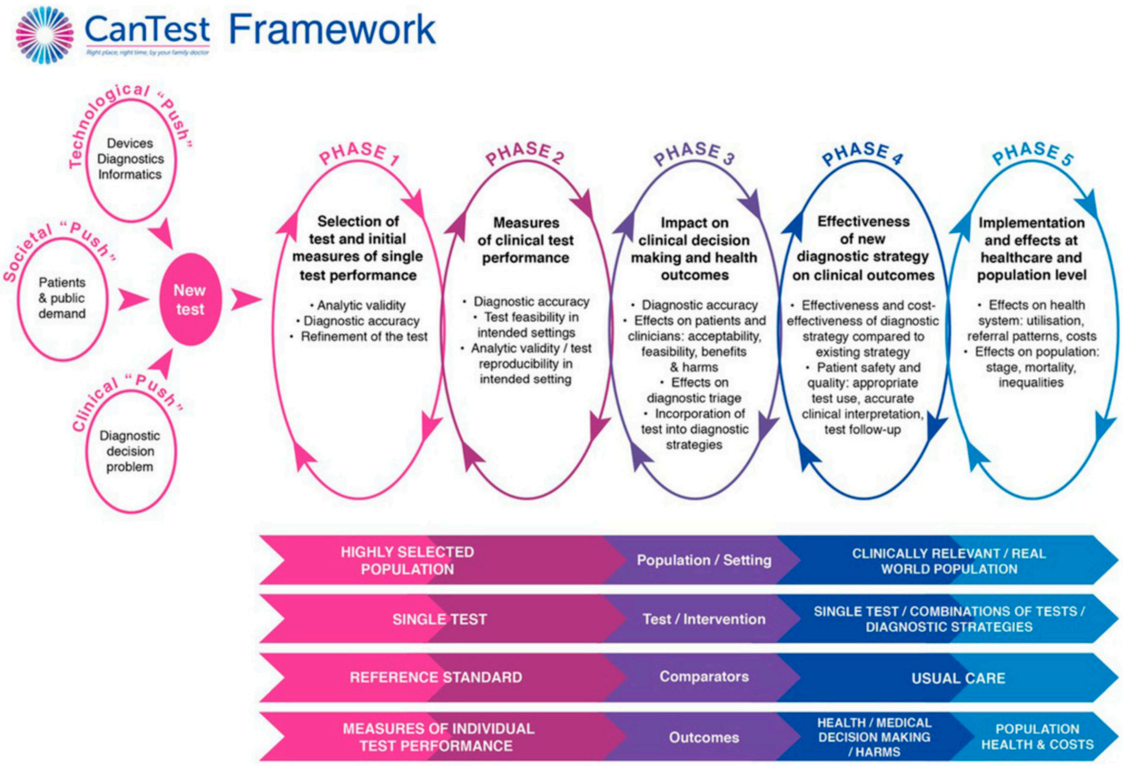

Walter FM; Thompson MJ; Wellwood I; Abel GA; Hamilton W; Johnson M; Lyratzopoulos G; Messenger MP; Neal RD; Rubin G; Singh H; Spencer A; Sutton S; Vedsted P; Emery JD. Evaluating diagnostic strategies for early detection of cancer: the CanTest framework. BMC Cancer 2019;19(1):586

Table S2. Ovid MEDLINE(R) and Epub Ahead of Print, In-Process, In-Data-Review & Other Non-Indexed Citations, Daily and Versions(R) 1946 to June 2, 2021*

1	exp Urinary Bladder Neoplasms/ or (bladder adj3 ((urete* or (urin* adj3 tract)) adj6 (cancer* or neoplas* or tumo?r* or carcinom*))).ti,ab.
2	((urete* or urethra or (urin* adj3 tract)) adj6 (cancer* or neoplas* or tumo?r* or carcinom*)).ti,ab.
3	exp ureteral neoplasms/
4	exp urethral neoplasms/
5	1 or 2 or 3 or 4
6	((biomarker* or bio-marker* or biological marker* or marker* or POC or POCT or point of care or point-of-care or rapid test* or bedside test* or near-patient test* or near patient test* or fingerprick or finger prick or non-invasive or noninvasive or non invasive or metabolite marker* or metabolomic* or metabolic* or metabolite* or microRNA* or miRNA* or miR or protein* or ctDNA or circulating tumor DNA or circulating tumour DNA or cell-free DNA or DNA methylation or methylated DNA or methylat* or antibod* or autoantibod* or auto-antibod* or volatile organic compound* or VOC* or volatolome* or antigen* or blood or blood-based or serum or sera or plasma or urin* or fecal or faecal or stool or breath or saliva* or test or testing or analy* or assay* or immunoassay* or panel* or screen* or microarray*) adj6 (diagnos* or detect*)).ti,ab.
7	exp early diagnosis/
8	exp Diagnosis/
9	7 or 8
10	exp Biomarkers/ or exp Point-of-Care Testing/ or exp Point of Care Systems/
11	9 and 10
12	6 or 11
13	(Sensitivit* or Specificit* or ROC Curve* or receiver operat* characteristic or ROC or predictive value* or PPV or NPV or false negative* or false positive* or true negative* or true positive* or accurac* or area under the curve* or AUC* or AUROC* or performance* or discriminat* ability or discriminat*).ti,ab.
14	exp ROC Curve/
15	exp diagnostic errors/ or exp false negative reactions/ or exp false positive reactions/
16	exp "Predictive Value of Tests"/ or exp "Sensitivity and Specificity"/
17	13 or 14 or 15 or 16
18	5 and 12 and 17
19	limit 18 to yr="2000 -Current"

Embase 1974 to 2021 June 1

1	exp *bladder tumor/ or (bladder adj3 ((urete* or (urin* adj3 tract)) adj6 (cancer* or neoplas* or tumo?r* or carcinom*))).ti,ab.
2	((urete* or urethra or (urin* adj3 tract)) adj6 (cancer* or neoplas* or tumo?r* or carcinom*)).ti,ab.

3	exp *urethra tumor/
4	exp *urinary tract tumor/
5	1 or 2 or 3 or 4
6	((biomarker* or bio-marker* or biological marker* or marker* or POC or POCT or point of care or point-of-care or rapid test* or bedside test* or near-patient test* or near patient test* or fingerprick or finger prick or non-invasive or noninvasive or non invasive or metabolite marker* or metabolomic* or metabolic* or metabolite* or microRNA* or miRNA* or miR or protein* or ctDNA or circulating tumor DNA or circulating tumour DNA or cell-free DNA or DNA methylation or methylated DNA or methylat* or antibod* or autoantibod* or auto-antibod* or volatile organic compound* or VOC* or volatolome* or antigen* or blood or blood-based or serum or sera or plasma or urin* or fecal or faecal or stool or breath or saliva* or test or testing or analy* or assay* or immunoassay* or panel* or screen* or microarray*) adj6 (diagnos* or detect*)).ti,ab.
7	exp *early diagnosis/
8	exp *cancer diagnosis/
9	exp *diagnosis/
10	7 or 8 or 9
11	exp *biological marker/ or exp *point of care system/ or exp *point of care testing/ or exp *bedside testing/
12	10 and 11
13	6 or 12
14	(Sensitivit* or Specificit* or ROC Curve* or receiver operat* characteristic or ROC or predictive value* or PPV or NPV or false negative* or false positive* or true negative* or true positive* or accurac* or area under the curve* or AUC* or AUROC* or performance* or discriminat* ability or discriminat*).ti,ab.
15	exp *receiver operating characteristic/
16	exp *"sensitivity and specificity"/
17	exp *diagnostic error/ or exp *false negative result/ or exp *false positive result/ or exp *missed diagnosis/
18	exp *area under the curve/
19	14 or 15 or 16 or 17 or 18
20	5 and 13 and 19
21	limit 20 to yr="2000 -Current"

*The same search string was used for updating searches to May 24th, 2022 (excl. time restrictions)

Figure S3. QUADAS-2: Summary of Results

QUADAS 2 - SUMMARY OF RESULTS

No	STUDY	RISK OF BIAS				APPLICABILITY CONCERNS		
		PATIENT SELECTION	INDEX TEST	REFERENCE STANDARD	FLOW & TIMING	PATIENT SELECTION	INDEX TEST	REFERENCE STANDARD
1	Attallah et al. 2015	?	☹	?	?	☺	☺	☺
2	Barbieri et al. 2011	☺	☹	☹	☹	☺	☺	☺
3	Bhuiyan et al. 2003	?	?	?	☹	☺	☺	☺
4	Critselis et al. 2019	☺	☹	☺	☹	☺	☺	☺
5	Dahmcke et al. 2016	☺	☹	☺	☹	☺	☺	☺
6	Davidson et al. 2020	?	☹	☹	☹	☺	☺	☺
7	Deininger et al. 2017	?	☹	?	?	☺	☺	☺
8	Dudderidge et al. 2020	?	?	?	☹	☺	☺	☺
9	Eissa et al. 2007	☹	☹	?	☹	☺	☺	☺
10	Eissa et al. 2007	☹	☹	?	☹	☺	☺	☺
11	Eissa et al. 2007	☹	☹	?	☹	☺	☺	☺
12	Eissa et al. 2009	☹	☹	?	☹	☺	☺	☺
13	Eissa et al. 2010	☹	☹	?	☹	☺	☺	☺
14	Eissa et al. 2011	☹	☹	?	☹	☺	☺	☺
15	Eissa et al. 2012	☹	☹	?	☹	☺	☺	☺
16	Eissa et al. 2014	☹	☹	?	☹	☺	☺	☺
17	Eissa et al. 2014	☹	?	?	☹	☺	☺	☺
18	Fu et al. 2018	?	?	☺	?	☺	☺	☺
19	Grossman et al. 2005	?	☺	☹	?	☺	☺	☺
20	Horstmann et al. 2012	?	☹	?	☹	☺	☺	☺
21	Karnes et al. 2012	☹	☹	☹	☹	☺	☺	☺
22	Kelly et al. 2012	☺	?	☺	☹	☺	☺	☺
23	Liu et al. 2016	?	☹	☹	?	☺	☺	☺
24	Meiers e al. 2007	?	☹	?	☹	☺	?	☺
25	O' Sullivan et al. 2012	?	?	☹	☹	☺	☺	☺
26	Oertl et al. 2007	☹	☹	?	?	☺	☺	☺
27	Piaton et al. 2003	☺	?	☹	☹	☺	☺	☺
28	Poulakis et al. 2001	☹	☹	☺	☺	☺	☺	☺
29	Saad et al. 2002	?	?	?	☹	☺	☺	☺
30	Sajid et al. 2020	?	?	?	?	☺	☺	☺
31	Sanchez-Carbayo et al. 2000	☹	☹	?	?	☺	☺	☺
32	Sarosdy et al. 2006	☹	☹	☹	☹	☺	☺	☺
33	Shang et al. 2021	?	☺	?	☹	☺	☺	☺
34	Todenhöfer et al.2013	☹	☹	?	☹	☺	☺	☺
35	Todenhöfer et al. 2013	☹	☹	?	☹	☺	☺	☺
36	Todenhöfer et al. 2013	?	☹	?	☹	☺	☺	☺
37	van Kessel et al. 2016	?	☹	?	☹	☺	☺	☺
38	van Kessel et al. 2017	☹	?	☹	?	☺	☺	☺
39	van Kessel et al. 2020	☹	☹	☹	☹	☺	☺	☺
40	van Valenberg et al. 2021	☹	?	☹	☹	☺	☺	☺
41	Virk et al. 2017	☹	☹	☺	☹	☺	☺	☺
42	Ward et al. 2022	☺	?	☹	?	☺	☺	☺
43	Wu et al. 2020	☹	☹	?	☹	☺	☺	☺
44	Zhou et al. 2019	☹	☹	☹	☹	☺	☺	☺

LOW	☺
HIGH	☹
UNCLEAR	?

Figure S4. Forest plots of sensitivity and specificity for NMP-22, UroVysion, uCyt+, BTAstat and FGFR3

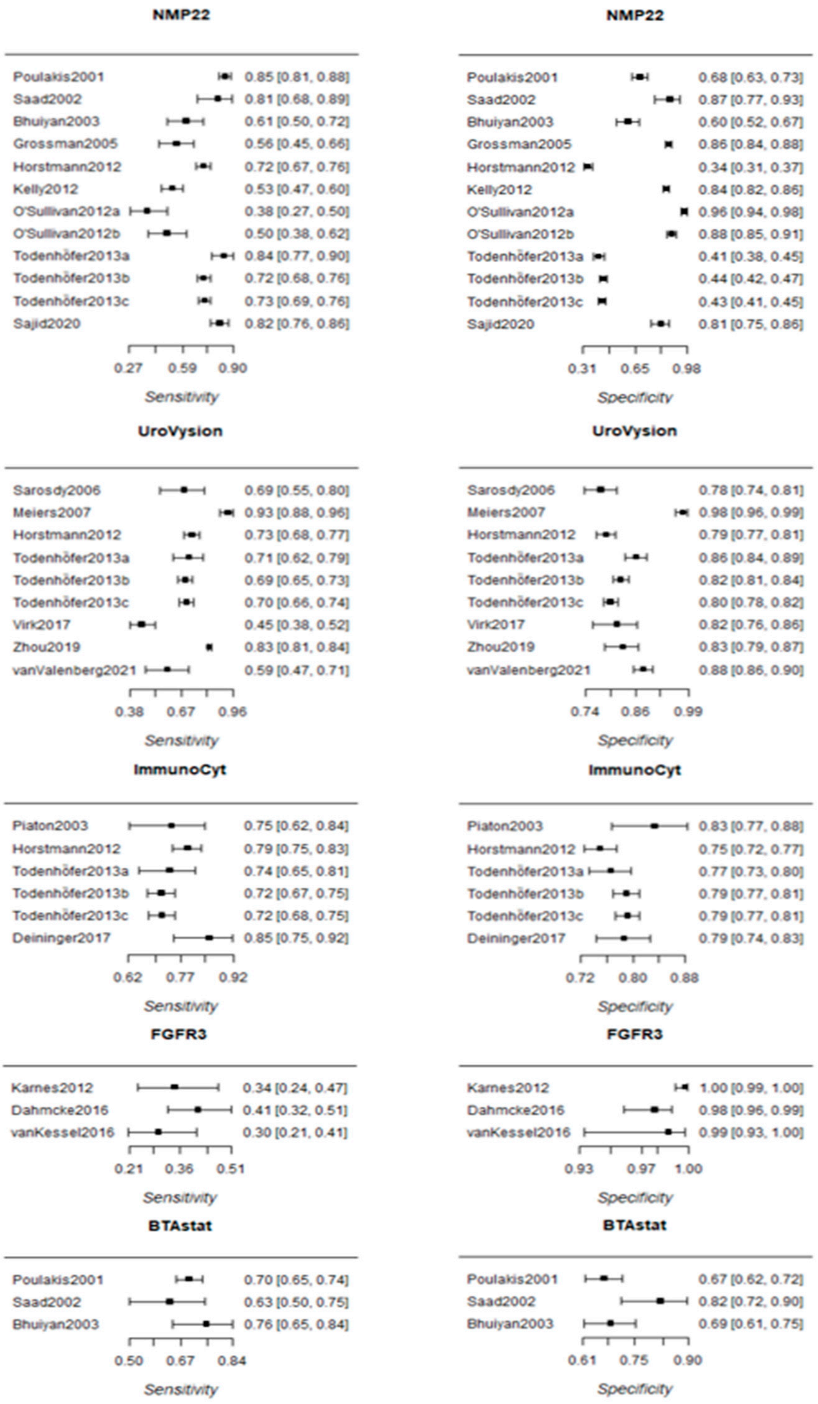

Figure S5. Sensitivity analysis for NMP-22 ELISA vs. BladderChek

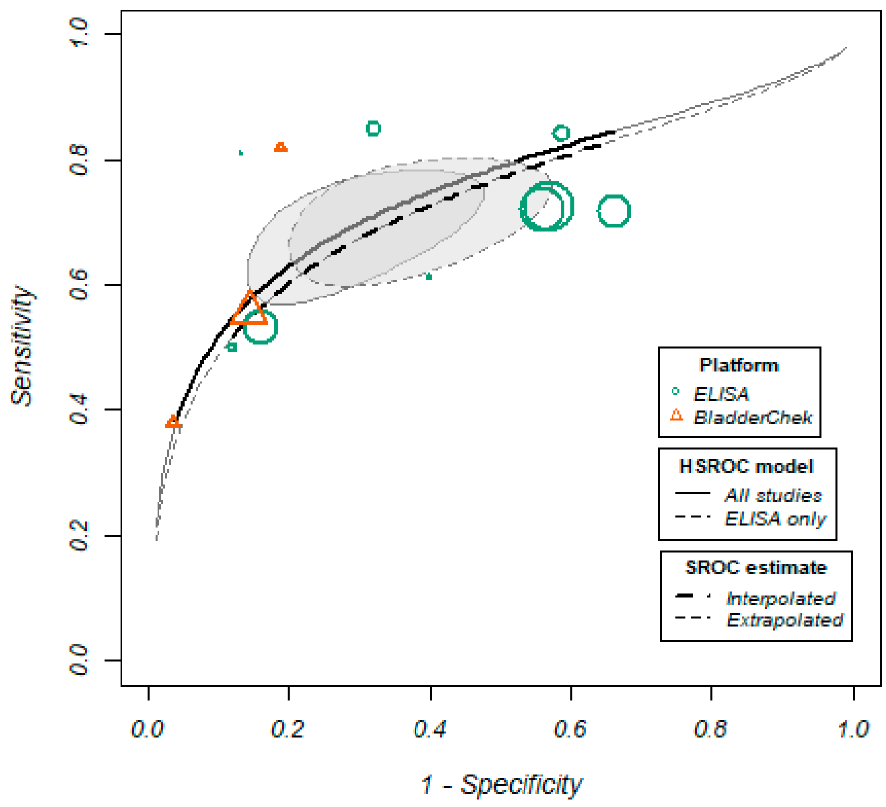

Supplement: Supplementary file 1 [file cancers-15-00709-s001.zip › cancers-2172443-supplementary.pdf]
